# Mutant p53-Associated Molecular Mechanisms of ROS Regulation in Cancer Cells

**DOI:** 10.3390/biom10030361

**Published:** 2020-02-26

**Authors:** Marco Cordani, Giovanna Butera, Raffaella Pacchiana, Francesca Masetto, Nidula Mullappilly, Chiara Riganti, Massimo Donadelli

**Affiliations:** 1 IMDEA Nanociencia, Ciudad Universitaria de Cantoblanco, 28049 Madrid, Spain; marco.cordani@imdea.org; 2 Department of Neurosciences, Biomedicine and Movement Sciences, Section of Biochemistry, University of Verona, 37134 Verona, Italy; giovanna.butera@univr.it (G.B.); raffaella.pacchiana@univr.it (R.P.); francesca.masetto@univr.it (F.M.); nidula.mullappilly@univr.it (N.M.); 3 Department of Oncology, University of Torino, 10126 Torino, Italy; chiara.riganti@unito.it

**Keywords:** mutant p53, Gain-of-function, ROS, oxidative stress, cancer

## Abstract

The *TP53* tumor suppressor gene is the most frequently altered gene in tumors and an increasing number of studies highlight that mutant p53 proteins can acquire oncogenic properties, referred to as gain-of-function (GOF). Reactive oxygen species (ROS) play critical roles as intracellular messengers, regulating numerous signaling pathways linked to metabolism and cell growth. Tumor cells frequently display higher ROS levels compared to healthy cells as a result of their increased metabolism as well as serving as an oncogenic agent because of its damaging and mutational properties. Several studies reported that in contrast with the wild type protein, mutant p53 isoforms fail to exert antioxidant activities and rather increase intracellular ROS, driving a pro-tumorigenic survival. These pro-oxidant oncogenic abilities of GOF mutant p53 include signaling and metabolic rewiring, as well as the modulation of critical ROS-related transcription factors and antioxidant systems, which lead ROS unbalance linked to tumor progression. The studies summarized here highlight that GOF mutant p53 isoforms might constitute major targets for selective therapeutic intervention against several types of tumors and that ROS enhancement driven by mutant p53 might represent an “Achilles heel” of cancer cells, suggesting pro-oxidant drugs as a therapeutic approach for cancer patients bearing the mutant *TP53* gene.

## 1. Introduction

Tumor cells exhibit several metabolic alterations to fulfill the energy needs of uncontrolled growth [1]. Alteration in redox signaling stimulating reactive oxygen species (ROS) production is one of the hallmarks of cancer progression [2]. ROS play an essential role as intra- and extracellular messengers. A common characteristic among the different types of ROS is their capacity to cause oxidative alterations to proteins, DNA, and lipids, playing a role at every stage of cancer development, including initiation, promotion, and progression [2]. Activation of oncogenes is one of the factors that contribute to high levels of ROS in cancer. Among the innumerable set of genetic mutations in cancer cells, one of the most frequently occurs in the *TP53* gene [3]. The primary consequence of *TP53* alterations is the loss of wild-type functions that deprive cells of p53 tumor suppressive roles, such as the stimulation of apoptosis and regulation of cell cycle [4]. In addition, some missense *TP53* mutations encode proteins with structural alterations, especially in the DNA binding domain (DBD) and generate mutant p53 isoforms showing new oncogenic ability, referred to as gain-of-function (GOF) [5]. Many years of research unveiled that GOF p53 mutations support tumor progression by regulating a complex overview of diversified pathways associated with: adaptive metabolic switch in responses to cancer-related stressing conditions; reduced response to chemotherapy; promotion of migration, invasion, and metastasis [6,7]. Cancer cells expressing mutant p53 show high levels of ROS compared with wild type p53 cells and we and others discovered that GOF mutant p53 isoforms, among the other abilities, contribute to enhance ROS levels in cancer cells through a coordinated regulation of several redox-related enzymes and signaling pathways, thus favoring cancer cell growth [8].

In this review, we summarize the critical role that mutant p53, contrarily to its wild-type counterpart, exerts on ROS production in cancer cells, providing an overview of the discovered molecular mechanisms. These observations stress the importance of novel and personalized therapeutic interventions for cancer patients carrying mutant *TP53* gene in order to uncover new molecular targets to prevent the GOF mutant p53-driven alterations on cancer energy metabolism, which sustains tumor progression.

## 2. Reactive Oxygen Species: Types and Formation

ROS include radical and non-radical oxygen species formed by the partial reduction of molecular oxygen and are characterized by short-life and high instability. Free radicals, such as, for instance, superoxide ions (O_2_^•−^), contain unpaired electrons and are capable of independent existence. Instead, non-radicals can be oxidizing agents easily converted in radicals as the highly reactive compound peroxynitrite (ONOO^–^) [9].

The ROS origin is exogenous or endogenous. The endogenous formation occurs mainly in mitochondria by leakage of electrons from the electron transport chain (ETC) during cell respiration [10]. The exogenous formation, on the other hand, may be due to stressing factors in the external environment such as radiation, pollutant, or to certain xenobiotic compounds like cross-linkers and bacterial invasion [11].

In physiological conditions, ROS are involved in a wide range of cellular functions, acting mainly as second messengers in signal transduction of intra- and extracellular pathways to modify the redox state of proteins or lipids. In this way, ROS could modulate cell proliferation, differentiation, and maturation [12,13].

Different amounts of intracellular ROS lead to different cellular responses that could be changed in a dose dependent manner. At low levels, ROS play physiological functions as mentioned above, while at higher levels, when redox homeostasis fails, ROS may cause cellular dysfunctions and promote genomic instability, leading to neoplastic transformation or other pathological conditions, such as atherosclerosis, diabetes, neurodegeneration, and aging [14,15]. However, an excessive ROS increase leads to cell death following the damage of biomolecules and organelles essentials for cellular life [16,17,18,19].

Having a key role in many physio-pathological processes, ROS homeostasis is highly regulated by multiple antioxidant programs. In healthy cells, ROS balance is maintained by antioxidant defenses including non-enzymatic systems, such as glutathione and vitamins A and C, or antioxidant enzymes such as superoxide dismutases, catalase, and peroxiredoxins [20]. These molecules have been described to prevent oxidative damage acting as tumor suppressors and anti-aging systems [16].

However, upon cellular stresses, a dramatic increase in ROS levels can exceed the cellular capacity to counteract them. This condition is known as oxidative stress, in which the resulting ROS unbalance may be caused both by increase in new formation of ROS or a decrease in cellular capacity to maintain their homeostasis by antioxidant activities [18].

High levels of ROS can activate pro-survival pathways, leading to DNA damage, genomic instability, metabolic alterations, and drug resistance [19]. In cancer cells, intracellular ROS level undergoes a sophisticated regulation by multiple mechanisms (detailed in Section 2.1) and, therapeutically, their excess may sensitize tumor cells to chemotherapy [21]. In this regard, drug treatments usually trigger a further increase of ROS that may exhaust the cellular antioxidant capacity, thus leading to cancer cell death. This suggests novel therapeutic approaches based on ROS over-stimulation in cancer cells [22].

### 2.1. Main Antioxidant Enzymes Involved in Tumor Progression

Cells have developed several mechanisms of self-defense against oxidative stressors, most of them involving ROS-scavenging systems, such as superoxide dismutases (SODs). SOD is a protein family of three class of metalloenzymes localized in different compartments inside cells performing the same reaction but with different metal ions as cofactors. SOD1, or CuZn-SOD, is a copper and zinc-containing homodimer that is found almost exclusively in intracellular cytoplasmic spaces. SOD2, or Mn-SOD, is a manganese-containing tetramer located in mitochondrial spaces. SOD3, or EC-SOD, exists as a copper and zinc-containing tetramer, and is secreted exclusively to extracellular environment [23].

The key role of SOD family is demonstrated in many models, where its loss of activity is associated with an increased oxidative damage such as lipid peroxidation, protein carbonylation, and DNA breakage [24]. Mutations in the *SOD2* gene are associated to various types of cancer. Depending on the type of tumor and its progression, SOD2 may have contrasting effects [24]. Loss of SOD2 expression can favor cancer cell progression mediated by increasing ROS-mediated DNA alterations [25]. Since changes in SOD2 activity may regulate different stages of the cell cycle [26], a decrease in SOD2 expression may stimulate cell cycle progression mediated by O_2_^•−^ [27]. On the other side, an increased SOD2 activity may lead to tumor progression by modulating the levels of H_2_O_2_ in mitochondria [28].

Other scavenging enzymes, including catalase, glutathione peroxidases (GPXs), peroxiredoxins (PRXs), and thioredoxins (TRXs) may favor the reduction of H_2_O_2_, an uncharged species more stable than superoxide, to H_2_O [29]. In particular, the glutathione (GSH) system is comprised of different enzymes: glutathione reductase (GSR), glutathione peroxidase (GPX), and glutathione-s-transferase (GST) [30]. GSH directly reacts with ROS, generating less reactive conjugates [31]. In addition, GSH is used as a co-factor by GPX in the detoxification of H_2_O_2_ and ONOO^–^ [32]. In cancer cells, GSH represents a mechanism of chemoresistance. In fact, many chemotherapeutics are electrophilic compounds that are detoxified by GSH conjugation [33].

### 2.2. Main Redox-Related Signaling Pathways and Transcription Factors Involved in Tumor Progression

ROS are reported to take part in epigenetic processes of DNA methylation, histone methylation, and histone acetylation, leading to altered gene expression and genetic instability of several genes [34]. Furthermore, ROS can modulate the expression or activities of many transcription factors and proteins, including their stability [35,36]. Indeed, some critical transcription factors and signaling proteins can undergo to the oxidation of their redox-sensitive cysteine residues [29]. AKT is a proto-oncogene acting as anti-apoptotic factor, but upon ROS exacerbation, it may promote cancer cell death [37]. On the other side, AMP-activated protein kinase (AMPK) is an energy sensor activated upon stressing conditions to promote metabolic reprogramming and maintain redox balance [38,39]. AMPK can also be regulated by ROS and metabolic stress-induced ROS signaling affects the cross-talk between AMPK and AKT pathways [39,40].

Compared to normal cells, malignant cells favor higher levels of endogenous oxidative stress which influence signal-transduction pathways to stimulate cell growth and adaptive responses [41,42,43]. Indeed, in order to maintain cell survival, ROS interacts directly with critical molecules, such as NF-E2-related factor 2 (Nrf2) and sestrins (SESNs), to initiate signaling involved in various cellular processes. In normal conditions, Nrf2 is ubiquitinated and degraded by its association with Kelch-like ECH-associated protein-1 (Keap1) [44]. In tumor cells, Nrf2, increases the expression of antioxidant proteins in order to maintain redox balance. Moreover, Nrf2 is stabilized by Keap1 oxidation on its sensitive cysteine resulting in nuclear translocation, where it increases the expression of crucial antioxidant genes [19].

### 2.3. Main Non-Enzymatic Antioxidant Systems Involved in Tumor Progression

Redox homeostasis is also regulated by non-enzymatic proteins as SESNs or mitochondrial uncoupling proteins (UCPs). SESNs are a family of highly conserved stress-inducible proteins that are strongly up-regulated by oxidative stress as an antioxidant adaptive response [45,46]. During oxidative stress, their expression may be controlled from induced transcription factors, such as p53, Nrf2, or FOXO. Indeed, a positive feedback activation of Nrf2 results in an increased expression of the scavenging enzyme sulforedoxin (SRX) mediated by SESNs to orchestrate the antioxidant defense [47]. A recent report demonstrated that SESN2 can inhibit the pro-oxidant enzyme NADPH oxidase 4 (NOX4), preventing pathogenic amounts of cytosolic ROS [48]. SESNs may also contribute to redox homeostasis through the regulation of AMPK-mTORC1 signaling pathways, preventing mammalian target of rapamycin complex 1 (mTORC1) hyperactivation [49] which may enhance ROS production via inhibition of autophagy [50] or directly acting on mitochondrial function [46]. SESN-dependent inhibition of mTORC1 can be also important for autophagy-mediated degradation of Keap1, the inhibitor of Nrf2 [46].

UCP proteins, a family of five members in mammals, also has a key role as redox sensor localized in the inner mitochondrial membrane. The role of UCPs in tumorigenesis and cancer growth has recently been a focus for many researchers. In particular, UCP2 has been strongly related to tumorigenesis since it is involved in cancer cell proliferation and chemoresistance [51]. In cancer cells, UCP2 is generally overexpressed to attenuate ROS formation and to trigger anti-apoptotic properties and resistance to therapies [51]. On the other side, in early phases of tumorigenesis, UCP2 is down-regulated to allow ROS increase and genomic instability [52,53]. Interestingly, UCP2 can remodel the metabolism toward the Warburg effect, by shifting cancer cells metabolism from mitochondrial oxidative phosphorylation (OXPHOS) to aerobic glycolysis [53,54].

Moreover, UCP1 has been shown to be expressed in prostate cancer and be related to a metastatic phenotype. A study in skin cancer suggested that UCP3 increases lipid oxidation but limits tumorigenesis through the inhibition of Akt [55]. Alternatively, increased UCP3 expression has been reported in gastrointestinal adenocarcinomas [56] and in renal cell carcinoma [57]. More recently, Giatromanolaki et al. showed that UCP3 was significantly related to poor prognosis in squamous cell carcinomas. Interestingly, UCP1 and UCP3 are overexpressed in a large subgroup of non-small cell lung tumors and their expression coincides with increased glucose absorption, intensified glycolysis, and anaerobic glucose usage [58].

## 3. Structure and Function of the Tumor Suppressor p53

The p53 protein is encoded by the oncosuppressor *TP53* gene located on 17p13.1 chromosome. It acts as a transcription factor consisting of 393 amino acids and containing four functional domains: (i) N-terminal region (TAD—transactivating region), which regulates transcription by interacting with different transcription factors such as TATA Binding Protein (TBP); (ii) proline rich-regions (aa 63-97), which is required for p53 stabilization; (iii) a central region of DNA binding domain (DBD), which binds to specific sequences present on regulated gene promoters; (iv) C-terminal region, which has two partially overlapping domains, one of which recognizes and binds to the damaged DNA (TET region) [59]. The DNA sequence that binds p53 (P53RE = P53 responsive element) consists of two palindromic sequences, one of the two DNA filaments, therefore including four similar sites, each able to bind to a p53 monomer [60]. Thus, p53 binds DNA and activates gene transcription in a homotetrameric conformation [59]. Indeed, the tumour suppression activity exercised by p53 is mainly due to its transcriptional activity. Many p53 target genes contain one or more P53REs in their promoter, inducing the expression of a large number of genes that encode proteins involved in multiple biological responses, such as DNA repair, cell proliferation, cell cycle distribution, and eventually apoptosis. Since p53 prevents cell mutations by promoting the DNA damage repair in injured cells, it is also called “guardian of genome” [61,62]. P53 is also able to regulate gene expression indirectly by regulating the activity of other transcriptional factors or stimulating the expression of some specific microRNAs [63]. Many studies revealed that p53 mediates tumor suppressor function, leading an antioxidant response to maintain genome integrity and to prevent the activation of oxidative stress-activated signaling pathways leading cell proliferation, such as PI3K-Akt and mTOR [64]. On the other side, enhanced ROS generation deriving by chemotherapy treatments was found lead both apoptosis and DNA repair in a p53-dependent manner [65,66], suggesting the existence of multiple pathways that integrate oxidative stress and p53 signaling. In addition, upon oxidative stress, mitochondrial translocation of p53 drives changes in mitochondrial membrane potential, cytochrome c release, and caspase activation [67,68].

The p53 is subjected to post-translational changes, such as phosphorylation and acetylation, that are crucial modifications for the regulation of its activity. P53 can also undergo ubiquitination and proteolytic degradation by the proteasome, limiting the p53 half-life and its intracellular accumulation. This regulation depends on a negative feedback mechanism in which MDMs proteins are involved [69,70]. In normal cells, without damage capable of causing the activation of p53, the p53 half-life is very short and its intracellular levels are very low [71]. Intriguingly, ROS may also affect the stability and the activity of p53 through several post-translational modifications. In this sense, ROS have been implicated in the phosphorylation of p53 mediated via p38α MAPK (mitogen activated protein kinase) [72], ATM (ataxia-telangiectasia mutated protein) [73], and ERK (extracelluar signal-regulated kinases) [74]. Besides, p53 itself is a redox-sensitive protein that functions as a cellular redox sensor because of the presence of conserved cysteine residues containing redox-sensitive thiol groups. In this regard, Bezek et al. reported that oxidation of Cys 277 decreases p53 binding to GADD45, affecting DNA damage response [75], hypothesizing that redox modification of p53 might also represent a potential mechanism for selective gene regulation.

### P53 Mutations In Cancers: Gain-of-Function

The *TP53 gene* has an unusual mutational pattern. Indeed, the gene is not frequently deleted but is mainly subject to mutations, the majority of which are missense mutations located in the DBD. Indeed, mutations of *TP53* are harbored in all coding exons, but predominantly cluster in exons 4–9 corresponding to the DBD. Within this region, the most frequent mutations, known as hotspots, are divided into two categories: conformational mutations that lead to structural changes in the binding domain, and contact mutations, that alter the ability of the protein to bind DNA [76]. In both cases, these mutations alter the interaction of p53 with its consensus DNA-binding sequence, impairing the activation of p53 target genes involved in suppressing tumor growth. In almost all cancers, six ‘hotspot’ residues are frequently mutated in the *TP53* DBD, namely R175 (4.8%), G245 (3.12%), R248 (6.79%), R249 (2.59%), R273 (6.55%), and R282 (2.59%) (IARC *TP53* Database) [77]. Very high levels of mutant p53 proteins accumulate in tumors because of their inability to induce MDM2 expression. However, p53 mutants have not only lost the tumor suppressor function of wild type p53 associated with its transcriptional activity, but also exert a dominant negative effect on the co-expressed wild type protein. Indeed, mutant p53 heterodimerizes with wild type p53 to form complexes that impair its function. p53 mutations are usually followed by the deletion of the remaining wild type *TP53* allele. This phenomenon, known as loss of heterozygosity (LOH), suggests that despite the dominant negative effect exerted by p53 mutants, the complete loss of wild type p53 provides cancer cells with a selective advantage. Indeed, according to a recent in vivo study, p53 LOH is required for mutant p53 stabilization, and the execution of additional oncogenic functions. Indeed, mutant p53 proteins can also acquire novel pro-oncogenic properties, an effect known as gain of function (GOF).

In several human tumors, specific *TP53* mutations lead to poor prognosis. This can be better studied, with the example of patients suffering from Li-Fraumeni syndrome (LF), where germline missense p53 mutations have been associated with earlier age of tumor onset when compared to germline *TP53* loss [78]. Mutant human p53 alleles (p53-Ala143, p53-His175, p53- Trp248, p53-His273, and p53-Gly281) expressed in cell lines lacking wild type p53 resulted in either enhanced tumorigenic potential in nude mice or enhanced plating efficiency in agar cell culture [79].

Although the number of regulatory pathways of p53 are shared between wild type and mutant p53, a wide range of key differences account for the chronic stabilization and activation of mutant isoforms [80]. In the transgenic mouse model developed with the murine mutant p53-His172 under the control of human keratin-1-based Vector, some authors exhibited increased susceptibility to chemical carcinogenesis with greatly accelerated benign papilloma formation, malignant conversion, and metastasis [79]. By this attained gain of function by the mutation in the *TP53* gene, some other genes are also activated. They include proliferating cell nuclear antigen (PCNA) gene, epidermal growth factor receptor (EGFR), bFGF, insulin like growth factor receptor (IGF-1R), interleukin 6, VEGF, C-myc, MDM2, and others [79]. Also, numerous in vitro and xenograft models have confirmed the ability of mutant p53 isoforms to drive invasion and motility of cancer cells, with evidence that mutant p53 can enhance signaling through receptors such as transforming growth factor beta (TGF-β) receptor, EGFR, and MET receptor tyrosine kinase (RTK) [81]. The GOF acquired by mutant p53 is also supported by studies that demonstrate that patients carrying a *TP53* missense mutation leading to expression of a mutant p53 protein in the germline have a significantly earlier cancer onset than patients with mutations in *TP53* gene that result in loss of p53 protein expression [81].

In conclusion, the reactivation of the p53 wild type-related functions of mutant p53 isoforms, for instance by using molecular chaperones, or the elimination of mutant p53 expression/activity, can be considered a therapeutic strategy to reverse back the mutant p53 oncogenic GOFs.

## 4. Reactive Oxygen Species (ROS)-Related Mechanisms Regulated by Mutant p53

**Figure 1:**
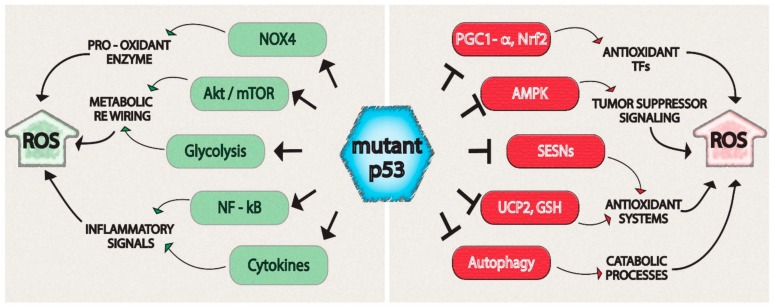
Gain-of-function mutant p53 orchestrates an oncogenic program that induces high levels of reactive oxygen species (ROS) to promote tumor progression.

A number of studies report that GOF mutant p53 proteins drive tumor progression by sustaining an oncogenic oxidant intracellular environment through a sophisticated coordination of signaling pathways and of redox-related enzymes. In the next section, we deeply describe the most significant molecular mechanisms by which mutant p53, contrarily to the wild type protein, regulates ROS production in cancer cells, giving result to new therapeutic opportunities. These mechanisms are summarized in Figure 1. The specified target molecules and biological process shown in Figure 1 are regulated by several mutant p53-isoforms, which are listed in Table 1.

### 4.1. Regulation of Sestrins (SESN)/AMP-Activated Protein Kinase (AMPK) Axis by Mutant p53

As commented above, SESNs and AMPK signaling are stress-inducible systems up-regulated by oxidative stress and their expression is strictly associated to the antioxidant adaptive response of the cells [45,82,83]. The antioxidant activity of SESNs depends on the reduction of PRXs and Nrf2 activation [47], as well as by the binding and stimulation of AMPK through upstream kinases, such as LKB1 [84,85]. Notably, AMPK signaling triggers the autophagy process and stimulates Nrf2 and PGC-1α-dependent (peroxisome proliferator-activated receptor gamma coactivator 1-alpha) antioxidant responses, exerting a critical role in mitochondria quality control [86,87], thus limiting mitochondrial ROS production [83,88]. Indeed, AMPK-mediated phosphorylation might modulate the ability of PGC-1α to dock on certain transcription factors or affect the binding or function of other cofactors in the PGC-1α coactivator complex. Therefore, both SESNs and AMPK responses participate in a functional axis that maintains metabolic and redox homeostasis in the cells and its alteration leads increasing oxidative stress and tumor progression [46,83].

Recently, some studies described that, in contrast to the antioxidant role of wild type p53, mutant p53 proteins can sustain ROS production, thus promoting chemoresistance and hyper-proliferation of cancer cells. However, the precise molecular mechanisms involved in this abnormal regulation of ROS by mutant p53 isoforms were still incomplete. In our previous studies, we reported that mutant p53 proteins (p53-R175H, p53-R248H, and p53-R273H) were able to repress the transcription of SESN1 and SESN2, and consequently the amount of the SESN/AMPK complex, resulting in the inhibition of AMPK signaling in pancreatic and breast cancer cells [8,89]. Notably, SESN/AMPK blockage has been shown functionally involved in the pro-oxidant role of mutant p53 in cancer cells through stimulation of mitochondrial O_2^−^_· production without damaging mitochondrial DNA (mtDNA) [8]. Moreover, through dysregulation of SESN/AMPK, axis mutant p53 proteins have been shown to promote autophagy defects in cancer cells [89], which may lead in turn to accumulation of abnormal mitochondria and consequently ROS induction, genomic instability, and cancer initiation and progression [2]. Interestingly, the low expression levels of SESN1, as well as of other autophagy and ROS-related genes, strongly correlated with reduced relapse free survival (RFS) and distant metastasis free survival (DMFS) of breast cancer patients carrying *TP53* gene mutations conferring a prognostic value to mutant p53 and SESN1 expression [89].

Since SESN/AMPK axis is a master energy sensor and a crucial regulator of oxidative stress, the demonstration that different mutant p53 proteins are actively involved in the sustaining of cell growth, chemoresistance and regulation of oxidative stress through direct inhibition of this tumor suppressor axis provides novel mechanisms underlying the molecular aspects of GOF mutant p53 in cancers. In contrast to the known AMPK signaling activation by wild type p53, the inhibition of AMPK signaling by mutant p53 isoforms has a relevant mechanistic-translational implication in cancers. In this case, p53 mutation transforms a signaling network with tumor-suppressing functions into a network with oncogenic potential that leads oxidative stress to promote tumor growth and progression.

### 4.2. Regulation of Akt/mTOR Signaling by Mutant p53 Proteins

mTOR coordinates eukaryotic cell growth and metabolism with microenvironmental stimuli, including nutrients and growth factors. Extensive research during the last decades has attributed a crucial role for mTOR in the regulation of fundamental cellular processes as protein synthesis, autophagy, and oxidative stress and demonstrated that deregulated mTOR signaling is implicated in cancer progression and aging [90].

mTOR is a serine/threonine protein kinase belonging to the phosphatidylinositol kinase related kinase (PIKK) family and forms the catalytic subunit of two distinct protein complexes, known as mTORC1 and mTORC2 [91,92]. mTORC1 plays a central role in regulating anabolic pathways essential for cell growth, such as the production of proteins, lipids, and nucleotides while also suppressing catabolic pathways and autophagy [90,93].

Behind the regulation of cell growth, increasing evidence suggest that mTORC1 can be also involved in the regulation of oxidative stress and intracellular ROS levels.

Some evidence suggests that the reduction in mRNA translation during mTORC1 inhibition delays aging by reducing oxidative stress, consistent with the observation that loss of the mTORC1 substrate S6K1 also extends life span in mammals [94]. A related possibility is that inhibition of mTORC1 slows aging by increasing autophagy, which helps clear damaged proteins and mitochondria, the accumulation of which is also associated with increased oxidative stress and aging-related diseases [90].

In this sense, several studies attributed to mTORC1 a fundamental role in mitochondria turnover and activity by regulating ERR-α and PGC1-α target genes and through 4E-BP-dependent translational regulation, resulting in increased oxidative phosphorylation and mitochondrial ROS production [95,96,97].

Interestingly, it has been reported that the loss of the tuberous sclerosis complex genes (TSC1 or TSC2) leads to constitutive activation of mTOR and downstream signaling elements, resulting in endoplasmic reticulum (ER) stress [98,99], an important source of ROS generation and oxidative stress with multiple pathological implications [100,101,102,103].

An increasing amount of evidence highlights that oncogenic mutant p53 proteins, contrary to their wild type counterpart, can stimulate mTOR signaling, altering the redox balance and sustaining an oxidative environment where cancer cells can proliferate and grow. In this sense, Agarwal et al. reported that the ectopic expression of three hot-spot mutant p53 proteins, as R175H, R248W, and R273H, led the hyper-phosphorylation of the mTORC1 targets S6K1 and 4EBP1 in both p53-null HCT116 colon carcinoma cells and H1299 non-small cell lung carcinoma, contrarily to wild type p53 that reduced mTORC1 activity, as revealed by the decreased phosphorylation of S6K1 and 4EBP1 [104]. In a recent work, we demonstrated that mutant p53 proteins sustain mTOR signaling, which was able to suppress Beclin-1 phosphorylation resulting in the inhibition of autophagy cell death, and enhancement of cell proliferation and chemoresistance [89]. Intriguingly, the stimulation of mTOR pathway represents an Achilles heel of cancer cells bearing mutant p53, making them susceptible to mTOR inhibitors [89].

Aberrant dysregulation of phosphoinositide 3-kinase and Akt (PI3K/Akt) pathway plays a key role in many hallmarks of cancer and contributes to increase ROS levels through direct modulation of mitochondrial bioenergetics and activation of NOX, or through the generation of highly reactive metabolic by-products, as superoxide ions and H_2_O_2_ [105,106,107]. Importantly, a number of studies demonstrated that mutant p53 stimulates PI3K/Akt activity, leading to a variety of oncogenic proprieties, such as cell survival, cell migration, metabolic re-wiring, and anoikis resistance [108,109,110].

In this context, Tan et al. reported that depletion of endogenous hot-spot R273H, R280K, and R280T mutant p53 isoforms downregulated Akt phosphorylation in MDA-MB-468, MDA-MB-231, and CNE-1 cancer cell lines, respectively, suggesting the involvement of mutant p53 isoforms in the promotion of Akt signaling [108]. Intriguingly, the authors showed a direct correlation between high expression of mutant p53 and strong phospho-Akt staining in primary human breast cancers, providing further evidence of the clinical relevance of mutant p53 proteins in the PI3K/Akt axis stimulation [108]. We also showed that R273H mutant p53 induces the phosphorylation of Akt, which favors the nuclear stabilization of the glycolytic enzyme GAPDH having a critical impact in anti-apoptotic and anti-autophagic effects driven by mutant p53 [110].

Moreover, several studies demonstrated that mutant p53 proteins may sustain tumorigenesis by activating a number of growth factor receptors implicated in the activation of PI3K/Akt signaling, including transforming growth factor beta receptor (TGF-βR) [111], EGFR [112,113], MET receptor tyrosine kinase (RTK) [114,115], and IGF-1R [109], through multiple mechanisms. For example, the hot-spot R273H mutant p53 has been shown to stimulate Akt phosphorylation to promote cell migration and breast cancer development by activating IGF-1R and EGFR through the suppression of miR-30a and miR-27a [109,110,111,112,113].

Overall, the studies reported above suggest that mutant p53 proteins may exert their pro-oxidant functions through a sophisticate regulation of PI3K/Akt and mTOR pathways in cancer cells. Consequently, the molecular switch towards anabolic metabolism to support the uncontrolled growth rate typical of cancer cells may lead to the generation of high levels of ROS, which may act as critical mediators for the oncogenic proprieties of mutant p53.

### 4.3. Regulation of Glycolytic Metabolism by Mutant p53

The main metabolic feature of cancer cells is the “Warburg effect”, a metabolic switch to sustain glycolysis even in aerobic conditions. This metabolic process converts glucose into lactate even in the presence of oxygen and with functional mitochondria, while normal cells predominantly use cellular respiration as an energy source [116].

Notably, one of the strongest effects of mutant p53 on the alteration of cancer metabolism concerns the metabolism of glucose. Indeed, mutant p53 has been reported to sustain glucose intake and hence the Warburg effect in tumor cells and in mice models [117]. Hernández-Reséndiz et al. showed that in contrast to cancer cells bearing wild type p53, in which oxidative phosphorilation is the predominant ATP source, cancer cells having p53R248Q mutation exhibited high dependence on glycolytic ATP under normoxic conditions [118].

Mutant p53 stimulates glycolysis by different ways. Specifically, it promotes the translocation of GLUT1 (glucose transporter 1) to plasma membrane, which is mediated by an activated RhoA/ROCK signaling [118]. In addition, mutant p53 stabilizes the cytosolic localization of the glycolytic enzyme GAPDH, preventing its nuclear transport by both stimulation of Akt and repression of AMPK signaling and is also associated with the formation of the SIRT1:GAPDH complex [110]. This event further supports glycolysis pathway, enhances lactate secretion, and confers sensitivity of cancer cells to metabolic drugs, as the glycolytic inhibitor 2-deoxyglucose.

Furthermore, AMPK negatively regulates the Warburg effect through inhibition of the hypoxia-induced factor 1 (HIF1) pathway [119] and mutant p53s have been shown to promote HIF1 expression [120], which is known to promote glycolysis. One of the activator of HIF1 is the M2 isoform of pyruvate kinase (PKM2) [119]. We showed that mutant p53 induces PKM2 in a mTOR-dependent manner [121]. Since PKM2 supports anabolic metabolism and PKM2 and is a crucial metabolic enzyme in the oncogenic mTOR-induced glycolysis [122], we hypothesized that mTOR/PKM2 pathway might contribute to the metabolic alterations in cancer cells bearing mutant *TP53 gene*.

Which is the relationship between the Warburg effect and ROS production? Pyruvate is the end product of glycolysis in normal cells and is a potent antioxidant, protecting normal cells from the effects of oxidative stress [123]. The extracellular secretion of lactate produced through the Warburg effect acts as a proinflammatory effect, since lactate facilitates the further secretion of proinflammatory cytokines that have been shown to increase ROS production, as described in the next paragraph. In addition, loss of pyruvate through reduction into lactate wastes a metabolite that is consumed as a substrate for Krebs cycle, depriving cancer cells from the antioxidant properties of Krebs cycle intermediates such as citrate, malate, and oxaloacetate [124,125].

Since ROS are essential second messengers, a pathological circle of ROS-stimulated glucose uptake and glucose-stimulated ROS production may be involved in cancer [126]. Indeed, oxidative stress has been shown to up-regulate GLUT1 expression, increasing GLUT1 protein and glucose transport activity [127].

In conclusion, since cellular glucose metabolism and mitochondrial ROS production are coupled by various signaling mechanisms, mutant p53 proteins may exert their pro-oxidant functions, stimulating several molecular mechanisms resulting from upregulated glycolysis. From a therapeutic point of view, the mutp53-dependent enhanced glycolysis might represent a potential personalized therapeutic target in human cancers carrying the mutant *TP53 gene*.

### 4.4. Regulation of Antioxidant Systems or Enzymes by Mutant p53 Proteins

As compared to wild type p53 functions, mutant p53 isoforms have been also shown to differently regulate various antioxidant cellular systems or enzymes. For instance, glutathione levels are differentially regulated in cancer cell lines carrying wild type or mutant p53 proteins. In this sense, glutathione was found increased in WT-p53 breast cancer cells and downregulated decreased in pancreatic cancer cell lines carrying mutp53. Therefore, the differential regulation of glutathione for wild type or mutant p53 cancer cell lines supports the pro-oxidant role of mutant p53 isoforms [128].

NOX4 proteins are the catalytic subunit of the NADPH oxidase complex that catalyzes the reduction of molecular oxygen to various ROS [129]. NOX4 is found to be differentially regulated by wild type and mutant p53. A number of studies reported that WT-p53 suppresses NOX4 expression and thereby ROS production, while tumor-associated mutant p53 proteins enhance its expression [130]. Recently, we showed that mutant p53 tightly regulates oxidative stress in melanoma cells, stimulating mitochondrial SOD2 expression and SOD2 activity by SIRT3-mediated deacetylation [131]. This event is functional to keep the intracellular ROS increase moderate, in order to promote cancer cell proliferation and survival. The increase of SOD2 levels might be due to the induction of key SOD2 regulators such as c-Myc or NF-κB, which have been previously demonstrated to be stimulated by mutant p53 [132]. This study could be nearly translated in the clinic, since cancer patients bearing mutant *p53 gene* may benefit from a pro oxidant therapeutic strategy targeting SOD2. Finally, it has been also reported that mutant p53 correlates with an increase in lipid peroxides and a decrease in the antioxidant activity of catalase (CAT) and of its downstream targets [128].

### 4.5. Regulation of ROS-Related Transcription Factors by Mutant p53 Proteins

Regulation of oxidative stress-related transcription factors represents another important oncogenic role of mutant p53, by which it promotes ROS production and/or reduces the antioxidant defenses.

Nrf2 is a critical transcription factor controlling antioxidant defenses. It has been reported that mutant p53 physically interacts with Nrf2 and this interaction is crucial to promote pro-survival oxidative stress responses regulating Nrf2-target genes [133]. In this sense, mutant p53 may favor the nuclear translocation of Nrf2, thus redirecting it to the antioxidant response elements (ARE) in the promoter of Nrf2-target genes. This determines upregulation of thioredoxins (TXNs) and repression of detoxifying enzymes, such as NAD(P)H quinone dehydrogenase 1 (NQO1) and heme oxygenase 1 (HMOX1), resulting in intracellular ROS accumulation [133,134]. Interestingly, Liu et al. demonstrated that mutant p53 proteins mediated the repression of the Nrf2 target gene SLC7A11, a component of the cystine/glutamate antiporter [135]. The inhibition of the system xC^-^/glutathione axis diminishes glutathione synthesis rendering mutant-p53 tumors susceptible to oxidative damage. Thus, GOF mutant p53 isoforms can contribute to enhance ROS levels in cancer cells even through the binding with transcription factors, altering the transcription of its targets.

Moreover, in contrast to the wild type p53 role, mutant p53 enhances ROS level by regulating another key component of gene transcriptional machinery, i.e., PGC-1α, which is a versatile transcriptional coactivator that promotes the expression of many antioxidant/detoxifying enzymes and leads mitochondrial biogenesis [136]. In this regard, it has been demonstrated that mutant forms of p53 can inhibit PGC-1α function [137]. Furthermore, we showed that the inhibition of PGC-1α by mutant p53 is linked to the downregulation of mitochondrial UCP2 expression, favoring a determining ROS increase [8].

### 4.6. Stimulation of Pro-Inflammatory Cytokines

A plethora of studies have demonstrated that the crosstalk between inflammatory and tumor cells plays a pivotal role for cancer development and, for that reason, inflammation is considered one of the hallmarks of cancer [138].

The major players recruited in tumor microenvironment during inflammation are represented by inflammatory cells and by several biochemical mediators belonging to the wide family of cytokines, such as chemokines (CC, CXC, XC, and CX3C), tumor necrosis factor alpha (TNF-α), interferon gamma (IFN-γ), interleukin 6 (IL-6), TGF-β, and interleukin 10 (IL-10), which drive tumor aggressiveness by stimulating cell proliferation and motility [139,140]. Interestingly, these cytokines have been shown to increase ROS production in many cellular systems and through several mechanisms they contribute to keep a chronic pro-inflammatory and oxidative microenvironment that sustains tumor progression [141,142,143,144,145,146,147,148].

Several studies reported that wild type p53 can exert its anti-oxidant function by hampering inflammatory process through the inhibition of the activity of the transcription factor NF-κB [149,150,151], a key regulator of chemokines expression and of chronic inflammation that sustains cancer initiation and progression [152]. Besides, wild type p53 can down-regulate directly the transcription of various CXC chemokines and IL-6 leading to inhibition of angiogenesis and cell motility [153,154,155].

In contrast to their wild type counterpart, mutant p53 actively reshapes the profile of cytokines and chemokines secreted by cancer cells, contributing to establish an inflammatory microenvironment that might sustain high ROS level and support cancer cell growth and dissemination [156]. Yeudall et al. discovered that several mutant p53 isoforms were reported to induce the secretion of pro-inflammatory chemokines, such as CXCL5, CXCL8, and CXCL12, correlating this event with increased cancer cell migration and invasion [157].

The ability of mutant p53 to establish an inflammatory tumor microenvironment is mainly dependent on a functional interaction with NF-κB. In fact, mutant p53 has been shown to promote an efficient and prolonged NF-κB transcriptional activity in various cancer cells stimulated with TNFα, resulting in immune cell infiltration and inflammation [158,159,160,161]. In this way, it has been demonstrated that the inflammatory stimulus led by mutant p53 increases the incidence of invasive colon carcinoma in a mouse model of chronic colitis [160].

Notably, mutant p53 can activate TNFα-induced NF-κB pathway through the transcriptional inhibition of the tumor suppressor DAB2IP, a cytoplasmic inhibitor of NF-κB and concomitantly hampering TNFα-induced activation of ASK1/JNK [162,163]. This dual effect orchestrated by mutant p53 increases the secretion of chemokines resulting in lymphocytes infiltration and severe inflammatory tumor microenvironment.

Interestingly, it has been also proposed that the GOF mutant p53 isoform R175H can activate the expression of the pro-inflammatory chemokine CXCL1 in pancreatic and colon cancer cells by direct binding of its promoter in a NF-κB-independent mechanism [164].

In another study, Bossi and colleagues revealed that both conformational and contact hotspot p53 mutants, R175H and R273H, respectively, were able to induce inflammatory signals by inhibiting the expression of a natural anti-inflammatory cytokine, the secreted interleukin-1 receptor antagonist (sIL-1Ra). This occurred through the protein–protein interaction between mutant p53 isoform and the transcriptional corepressor MAFF, and their binding to the sIL-1Ra promoter [165].

These studies support the existence of different mechanisms induced by mutant p53 proteins to modulate the expression of secreted inflammatory cytokines in order to sustain an inflammatory tumor microenvironment, thus potentially contributing to promote oxidative stress and increased cancer aggressiveness.

## 5. Conclusions

Several studies summarized here report that wild type p53 and its mutant isoforms regulate oxidative stress in opposite ways. Under physiological or low stressing conditions, wild type p53 suppresses ROS production to inhibit DNA-oxidation, mutagenesis, and oxidative stress-activated signaling pathways involved in cell growth, such as PI3K-Akt and mTOR [64]. Wild type p53 prevents ROS accumulation by several mechanisms by inducing the expression of many antioxidant enzymes, including SOD2 and GPx1 responsible for decomposition of superoxide ions (O_2_^−.^) or hydrogen peroxide (H_2_O_2_), respectively [166]. Wild type p53 also induces the expression of SESNs, the expression and activity of CAT to lead H_2_O_2_ counteraction, as well as many other targets involved in redox regulation [167,168,169,170].

Many studies clearly showed that p53 mutant proteins, contrarily to their wild-type p53 counterpart, are unable to provide antioxidant responses, sustaining a controlled increase in intracellular ROS. Indeed, several oxidative stress targets regulated by wild type p53 are contrarily modulated by mutant p53 to sustain hyperproliferation-related DNA damage and tumor progression. For instance, Boudreau et al. showed that the p53 status differentially influences NOX4 activity in lung and breast epithelial cells [130]. We revealed that mutant p53 inhibits AMPK/PGC-1α/UCP2 axis, while the endogenous basal level of wild type p53 is not able to regulate this pathway [8].

Furthermore, metabolic alterations are differentially regulated on the basis of the p53 status. Indeed, mutant p53 sustains the Warburg effect, which may increase ROS production as described before, in contrast with the anti-glycolytic role of wild type p53 [171].

It is interesting to note that some tumor-derived p53 mutants may retain the pro-survival activities of wild type p53. Specifically, Humpton et al. reported that mutp53-R248W limits ROS accumulation upon serine/glycine starvation, showing adaptive functions to support tumor development.

Besides acting as critical regulator of ROS levels in cancer cells, GOF of mutant p53 is in turn regulated by oxidative stress in a functional interplay that sustains tumor progression. In a study by Lozano and colleagues, it was reported that the stability of mutant p53 was enhanced by high intracellular ROS, since their counteraction also decreased mutant p53 protein expression, suggesting that the oncogenic phenotype resulted from stabilized mutant p53 may be overcome by a chemotherapy regimen aimed to counteract ROS [172].

Concluding, we would stress that mutant p53 sustains tumor progression and cancer growth by stimulating ROS production through a coordinated regulation of redox-related enzymes and signaling pathways. Intriguingly, ROS enhancement driven by mutant p53 might represent an “Achilles heel” of cancer cells carrying mutant *TP53 gene*, as revealed by the mutp53-dependent acquisition of cell sensitivity to H_2_O_2_ treatment [8]. From a therapeutic point of view, we can speculate that cancer cells expressing mutant p53 proteins can be significantly more sensitive to pro-oxidant drugs, as compared to the wild type counterpart, leading to the excess of cytotoxic ROS accumulation and cancer cell death. This might provide new therapeutic challenging to be further considered for clinical studies in cancer patients bearing the mutant *TP53 gene*.

## Authors Contributions

All authors contributed to the conception and design, writing, critical revision, and final approval of the article. M.C., G.B., and M.D. conceived the article, supervised the study and coordinated the writing of the manuscript. All authors have read and agreed to the published version of the manuscript.

## Figures and Tables

**Table 1:** List of mutant p53 isoforms regulating different molecular targets and biological process in various tumor types.

p53 Mutant Isoforms	Target/Biological Process	Tumor Type
R175H, R280K	NOX4	Lung and breast cancer [130]
R273H, R280K, R280T	Akt	Nasopharyngeal carcinoma, breast and pancreatic cancer [108,109,110]
R175H, R248W, R273H	mTOR	Colon carcinoma, lung carcinoma, pancreatic and breast cancer [89,104,121]
R175H, R248Q, R273H	Glycolysis	Lung carcinoma, breast and pancreatic cancer [110,117,118,120,121]
R175H, R248W, R273H	NF-kB	Lung, pancreatic, breast and colon cancer [158,159,160,161,162]
R175H, R281G, R273H	Cytokines	Lung, breast, pancreatic and colon cancer [157,164,165]
R175H, R273H	PGC1-α	Lung, colon and pancreatic cancer [8,137]
R175H, R280K, R273H	NRF2	Colon carcinoma, oesophageal adenocarcinoma, lung and breast cancer [133,134,135]
R175H, R273H	AMPK	Pancreatic and breast cancer [8,89,110]
R175H, R248H, R273H	SESNs	Breast and pancreatic cancer [8,89]
R175H, R273H	UCP2	Lung, pancreatic and breast cancer [8]
R175H, R273H	GSH	Oesophageal adenocarcinoma, pancreatic and breast cancer [128,135]
R175H, R273H	Autophagy	Lung carcinoma, pancreatic and breast cancer [89]
